# Early structured communication between general practitioner, sick-listed patient, and employer: Results and lessons learned from a pragmatic trial in the Capacity Note project

**DOI:** 10.1017/S1463423624000574

**Published:** 2024-11-28

**Authors:** Paula Nordling, Chioma Nwaru, Lena Nordeman, Ingmarie Skoglund, Maria E.H. Larsson, Cecilia Björkelund, Gunnel Hensing

**Affiliations:** 1 School of Public Health and Community Medicine, Institute of Medicine, University of Gothenburg, Gothenburg, Sweden; 2 Primary Health Care, Region Västra Götaland, Gothenburg, Sweden; 3 Department of Health and Rehabilitation, Institute of Neuroscience and Physiology, University of Gothenburg, Gothenburg, Sweden; 4 Research, Education, Development & Innovation, Primary Health Care, Södra Älvsborg, Region Västra Götaland, Sweden; 5 Department of Education, Research and Development Primary Health Care, Region Västra Götaland, Gothenburg, Sweden; 6 Centre of Research and Education, Region Värmland, Karlstad, Sweden

**Keywords:** Family medicine, mental health, primary health care, return to work, sick leave, stakeholder collaboration

## Abstract

**Background and Objective:**

Early and collaborative interventions are desirable to prevent long-term sick leave and promote sustainable return-to-work (RTW). The aim of this study was to evaluate if the use of the Capacity Note – a brief intervention promoting early and structured communication between general practitioners (GPs), patients, and employers – had an impact on length of sick leave in patients with common mental disorders (CMDs) in primary healthcare.

**Method:**

In a pragmatic trial, GPs at eight primary healthcare centres were randomized to provide the intervention or control and recruited eligible patients: employed women and men, 18-64 years, who visited a GP due to CMD and became or were (<4 months) full- or part-time sick-listed. Patients in the intervention group (n=28) used the Capacity Note in addition to usual care. Patients in the control group (n=28) received usual care. Outcomes of interest were time until full RTW, sick leave status at end of follow-up (17 months), number of sick leave episodes during follow-up, and number of sick leave days at 6, 12, and 17 months of follow-up.

**Results:**

The proportion of patients with full RTW at the end of follow-up was 79.2% in the intervention group and 84.6% in the control group. Time until full RTW was 102 and 90 days (median) in intervention and control group, respectively. We found no statistically significant differences between the groups for any of the outcomes.

**Discussion:**

Despite efforts to increase the number of participants, the study ended up with a small sample. This prohibited us from drawing any final conclusions about the effect of the intervention. Obstacles to recruitment of patients and use of the intervention are discussed.

## Introduction

Sickness absence due to mental ill-health is still increasing in many Western countries. Common mental disorders (CMD), including depression, anxiety disorders, and stress-related disorders, are the most common diagnoses (OECD, 2012). The risk of long-term sick leave and recurring sick leave episodes is high for these conditions, and it places a great burden on individuals, employers, healthcare, and society at large (OECD, 2012, Andersen, 2010, WHO, 2017). The evidence for the effectiveness of interventions to improve return to work (RTW) in patients with mental ill-health is inconclusive (Nieuwenhuijsen *et al.*, 2020, van Vilsteren *et al.*, 2015, Brämberg *et al.*, 2024). However, improved collaboration and communication between stakeholders have been increasingly emphasized as important for a successful sick leave and rehabilitation process (Loisel and Durand, 2005, Franche *et al.*, 2005, Maiwald *et al.*, 2014, Wynne-Jones *et al.*, 2010).

Interventions to support communication between sick-listed employees and different stakeholders have been developed in several settings (Karlson *et al.*, 2010, van Oostrom *et al.*, 2010, Tamminga *et al.*, 2012, Munir *et al.*, 2013, Amin *et al.*, 2017, Hoefsmit *et al.*, 2016) but none as brief and simple as would be ideal for the primary healthcare setting, nor designed for the specific needs of patients with CMD. Primary healthcare is characterized by a heterogeneous patient group with a wide array of diagnoses and with limited time (approx. 15–30 min) per patient. During this short consultation, the GP’s primary focus is the medical issue, while work-related matters and sickness certification tend to be less prioritized (Nordling *et al.*, 2020, Nordling *et al.*, 2022).

Determining a person’s capacity to work and need for sick leave is a complex matter, especially in conditions like CMD where symptoms are often hard to observe and verify, and the effect on functioning is both fluctuating and highly individual (Bertilsson *et al.*, 2013, Bertilsson *et al.*, 2018, Dewa *et al.*, 2015). Research suggests that physicians make their assessment mainly based on clinical findings and the patient’s story, while their inquiry about the patient’s work situation is limited (Nordling *et al.*, 2020, Wynne-Jones *et al.*, 2010). In addition, communication with the patient’s workplace is hard to achieve due to lack of time and channels for communication (Nordling *et al.*, 2020, Wynne-Jones *et al.*, 2010), and the risk of breaching integrity (Russell *et al.*, 2005, Wynne-Jones *et al.*, 2010). A survey among 4228 Swedish GPs showed that 9 out of 10 found contacts with sick-listed patients’ employers valuable for their work with sickness certification, but only 4 out of 10 had such contacts regularly (Nordling *et al.*, 2021).

This study was part of the Capacity Note project where a brief communication facilitator was developed to support early and structured communication about mental health and RTW between the general practitioner (GP), the patient (on sick leave due to CMD), and the patient’s employer (Nordling *et al.*, 2022). The presumed benefits of using the intervention were for GPs a facilitated assessment of work capacity and a better basis for the sickness certificate, for employers a better basis for decisions about work adjustments, and for patients a more active and individualised sick leave process. The aim of this study was to test whether this approach could work in the primary healthcare setting and whether it affected length of sick leave.

## Material and methods

### Study design

The study was designed as a non-blinded randomized pragmatic trial with a follow-up of 17 months. Pragmatic trials are used to test if an intervention works in real life and is performed in routine practice settings rather than under controlled and well-defined research conditions (Patsopoulos, 2011). This is a common approach in primary healthcare trials and was in line with the theoretical assumptions underlying the design of the intervention (see below). The extended CONSORT guidelines for pragmatic trials (Zwarenstein *et al.*, 2008) guided the reporting of this study.

### Setting and procedures

The universal sickness insurance scheme in Sweden covers all inhabitants. The first 14 days of sick leave among employed persons are covered by the employer (except for a reduction equivalent to one-day sick salary). From the 15^th^ day, the Swedish Social Insurance Agency (SSIA) administers benefits. The individual can certify him-/herself sick for 7 days. From day 8, a medical certificate, issued by a physician and stating a reduced capacity to work due to medical symptoms, disease, or injury, is needed. Sick leave can be issued for full- or parttime (25, 50, or 75%).

Patients with CMD are generally managed in primary healthcare, as is their sick leave (when needed). The primary healthcare is organized in a demand-supply organization. The demand side is public and tax-financed while the supply side can be either public or private. Patients enrol themselves to a primary healthcare centre (PHC) of their own choice. The primary healthcare is responsible for treatment and medical rehabilitation. The SSIA and the employer share the responsibility for vocational rehabilitation. The employer is responsible for making relevant workplace adjustments and facilitating the employee’s RTW. Swedish secrecy law prohibits the physician from contacting the employer without consent from the patient.

**Figure 1.**
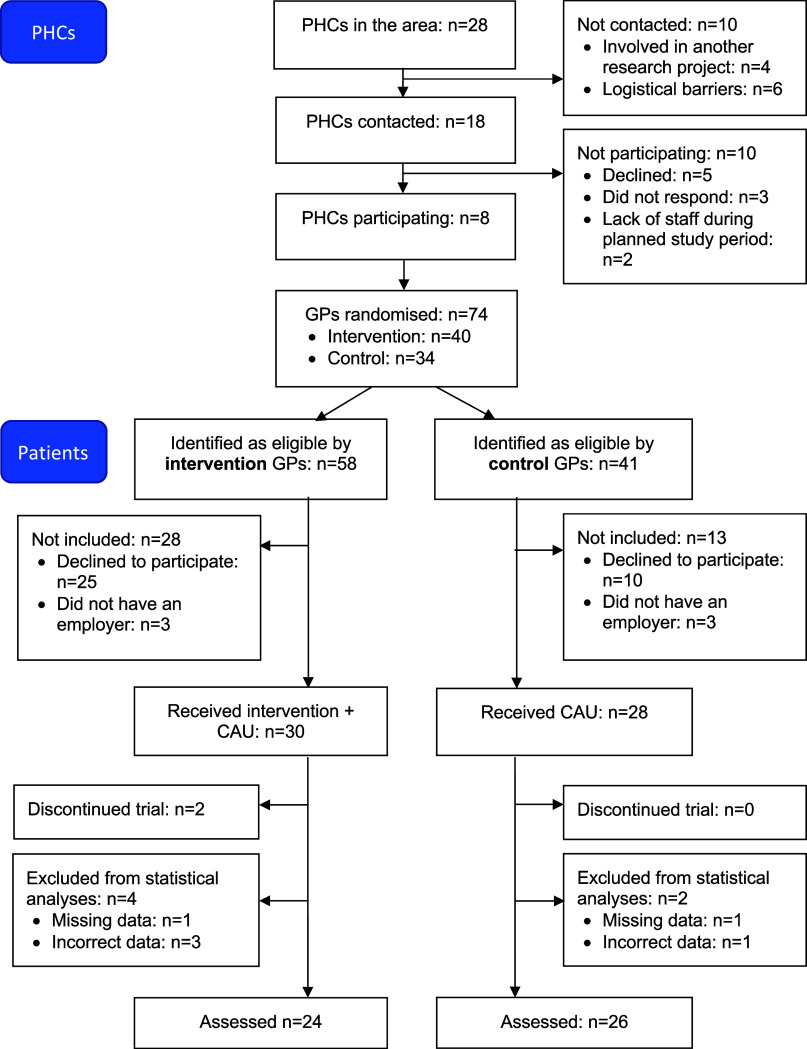
Recruitment and participant flow diagram. PHC = primary healthcare centre, GP = general practitioner, CAU = care as usual.

The study was carried out at public and private PHCs in the region of Västra Götaland in Sweden. There were several other primary healthcare research projects running in the region at the time and we were assigned a specific geographic area. Of the 28 PHCs in the area, four were involved in other research projects and six were considered too remote. The managers of the remaining 18 PHCs were contacted via e-mail with information about the study by either the head of the regions’ primary healthcare research and development centre (LN) or a GP active in both research and clinical work in the area (IMS). Five PHCs declined to participate and three did not respond. At the 10 PHCs that were interested, a meeting was held to further inform the PHC manager and the GPs about the study. These meetings were 30–60 minutes long, depending on what time span we were offered. All 10 PHCs accepted participation but due to a shortage of staff during the planned study period two could not participate (Figure 1). Characteristics of the eight participating PHCs are presented in Table 1. PHCs were financially compensated with 1100 SEK (approx. 100 EUR) per included intervention or control patient.

**Table 1.** Characteristics of the primary healthcare centres (PHCs) that participated in the study and their respective contributions

PHC	Town/ Rural area	Public/ Private	Participating GPs (n)	Study periods (n)	Weeks in the study (n)	Patients recruited (n)
1	Town	Public	7–10	2	4	6
2	Rural	Public	4–7	3	16	7
3	Rural	Public	6–7	2	6	11
4	Town	Private	7–9	2	5	9
5	Rural	Public	5–8	3	7	4
6	Rural	Public	9	1	4	4
7	Rural	Public	8	1	5	4
8	Rural	Public	2-8	2	18	13

Before the study started, all physicians handling sickness certification at each PHC were randomized to being intervention or control physicians. This was done manually by a research assistant not otherwise involved in the project. Specialists and those not yet specialists were randomized separately to ensure similar levels of experience among intervention and control physicians. Due to the design of the intervention, blinding was not possible.

At the study start, a brief meeting (5–30 min, again depending on what was offered) was held with the GPs for notification of randomization results, a repetition of study procedures, and distribution of written material such as the Capacity Note (see Intervention) and inclusion/exclusion criteria. GPs who were not present at these meetings were approached with this information later, e.g., during a lunch break. Both intervention and control physicians received information about the purpose, structure, and logistics of the intervention, but the full content was disclosed only to intervention GPs.

### Participants and recruitment

Eligible participants were employed women and men aged 18–64 years who consulted a GP due to common mental disorders (CMDs) and became part- or full-time sick-listed or had been so for no longer than four months. A current health status permitting decision-making about participation in the study was also required. Patients with severe psychiatric conditions, suicide risk, or severe somatic disease (e.g., cancer with secondary anxiety) were excluded.

Participating GPs were responsible for identifying eligible study participants during consultations and referring them to a research assistant who gave oral and written information and asked for informed consent. The research assistant was present at the PHC daily. After a few weeks, it became clear that many follow-up consultations concerning sick leave were telephone consultations and we added the possibility to recruit patients and use the intervention over the phone. Patients recruited this way were contacted by the research assistant after the consultation and then came to the PHC to give written consent.

### Intervention

**Figure 2.**
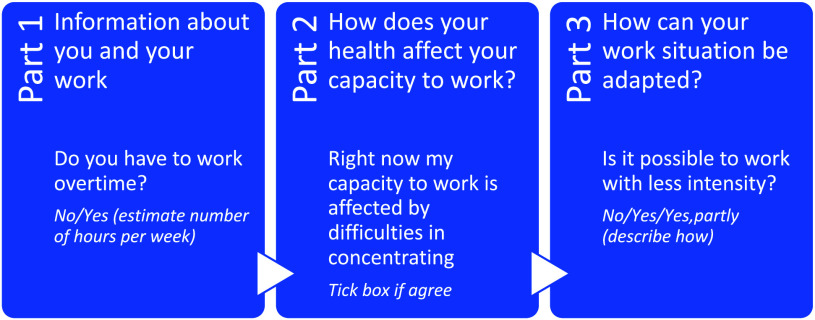
Examples of questions in each part of the Capacity Note.

The intervention consisted of a brief and structured way to discuss issues related to the patient’s current health and work situation using *the Capacity Note* – a paper form with three sections covering these topics; see Figure 2 for examples of questions in each section. The Capacity Note was used in three steps: 1) patient and GP discussing and filling in part 1 about work situation and part 2 about work capacity, 2) patient and employer discussing and filling in part 3 about work accommodations, and 3) follow-up at the next patient–GP contact. The intervention intended to facilitate a dialogue and information transfer between the stakeholders, thereby supporting their decision-making process in the sick leave and rehabilitation process. For more details, see below and in Nordling *et al.* (2022).

Theoretically, the intervention was inspired by the concept of *agency,* understood as the individual’s will and capacity to act independently (Bourdieu, 1977). Agency theory states that we accommodate social practice in relation to our position in a certain social context. Individual agency can be hampered by structural factors such as legislation, employment conditions, accessibility of information, and the social context at work (Ståhl *et al.*, 2021, Danielsson *et al.*, 2019, Bourdieu, 1977). Qualitative studies exploring patients’ experiences of becoming sick-listed suggest that after the initial emotional reactions a period of activity follows, which is later replaced by passivity and waiting for others to make decisions or take initiatives (Mårtensson and Hensing, 2012, Danielsson *et al.*, 2019). Agency can also be negatively affected by CMD symptoms such as worries, difficulties in making decisions and lack of energy and/or motivation (Bertilsson *et al.*, 2013, Danielsson *et al.*, 2017). Here, the GP has an important role in supporting the patient’s agency by providing treatment, information, and emotional support. The intervention was designed to strengthen the patient’s agency by making the patient more active in his/her sick leave process and by facilitating the GP’s assessment and subsequent recommendations. An important theoretical departure was to follow if patients managed to use the Capacity Note without too much involvement of clinicians or researchers.

#### Intervention group

In the intervention group, parts 1 and 2 of the Capacity Note were discussed and filled in by the patient and his/her GP during the consultation. The patient was then instructed by the GP to bring the Capacity Note to his/her manager for a joint discussion of part 3. The completed Capacity Note was to be sent back by the patient to the PHC, where it was added to the patient’s medical record and could be used for follow-up. There were no reminders or other contacts from the research team to influence the patients’ use of the Capacity Note. Neither did we check whether the Capacity Note was used for follow-up by the GP. In addition to using the Capacity Note, patients in the intervention group received care as usual. For patients with CMD in primary healthcare, this includes psychologist/counsellor contact, pharmacological treatment and/or referral to an occupational therapist, physiotherapist, and/or rehabilitation coordinator.

#### Control group

In the control group, patient and GP filled in part 1 of the Capacity Note during the consultation. This was done to enable comparison of patient demographics. The Capacity Note was then put aside, and the patient received care as usual (as described above).

### Data and outcomes

#### Participant characteristics

Data on age, sex, and occupation were collected from part 1 of the Capacity Note. Occupations were classified as skilled (> two years of college or university education) or unskilled (Statistics Sweden, 2012).

#### Sickness absence

Sick leave data were obtained from the Swedish Social Insurance Agency’s database Micro Data for Analysing the Social insurance (MiDAS). The database does not contain data on current employment but a reduction in or discontinuation of registered sickness absence days was used as a proxy for RTW. Outcomes of interest were time until full RTW, sick leave status at the end of follow-up, number of sick leave episodes during follow-up, and number of gross sick leave days at 6, 12, and 17 months after inclusion. A follow-up time of 17 months was chosen as it was considered long enough to reveal the sustainability of RTW but short enough to limit the effect of other factors than the intervention on the outcomes.

#### Feasibility

To assess the feasibility of the intervention, the following parameters were of interest: number of potentially eligible patients who were asked to participate, number of eligible patients who accepted participation, reasons for not participating, drop-out rate, and level of adherence to study protocol. This data were collected continually at each participating PHC using local sick leave registers and study logbooks. Patients who declined participation at either GP or research assistant were asked to provide (voluntarily) their age, sex, occupation, and reason for not participating.

### Statistical analyses

Descriptive statistics were used to summarize baseline characteristics of the study sample. Group differences in baseline characteristics, number of sick leave episodes during follow-up, and sick leave status at the end of the follow-up were analysed with the Chi-squared test or Fisher’s exact test, as appropriate. Full RTW was defined as having no registered sickness absence for at least four weeks. Time until full RTW, measured as number of days from date of inclusion in the study to full RTW, was calculated using Kaplan–Meier survival analysis and survival curves for the groups were compared with the log-rank test. The number of gross sick leave days at 6, 12, and 17 months of follow-up was calculated using the Mann–Whitney U test since the data during these follow-up times was not normally distributed. Only sickness absence due to CMD was analysed. Statistical significance was considered as a two-sided p-value <0.05. All statistical analyses were performed using Stata version 16 and IBM SPSS Statistics version 26.

## Results

### Recruitment

Data collection started in March 2018 and ended in Sept 2019 due to time constraints. Initially, the study periods were short, 2–5 weeks, and the study ran at one PHC at a time. In the end, to increase recruitment, the study ran continuously at two of the PHCs for 3 and 5 months respectively but with only a limited number of GPs active in recruiting patients.

To roughly estimate the number of potentially eligible patients, each participating PHC’s local sick leave register was checked at the start of their respective study period. Of the total number of sick leave cases at each PHC, 55% (mean) were due to CMD and of these, 37% (mean) were shorter than 4 months. The pattern was similar at all PHCs and can be illustrated with the numbers from PHC 7: a total of 145 patients were on sick leave, 72 cases were due to CMD, and 26 cases were shorter than four months. Considering in- and outflow, the total number of potentially eligible patients during the study (65 weeks) was calculated to be about 500 patients. The GPs identified 99 patients as eligible for participation. Of these, 64 accepted participation: 33/58 (57%) in the intervention group and 31/41 (76%) in the control group (Figure 1). Reasons for not participating are presented in Table 2. Six patients were excluded as they did not have an employer. In total, 58 patients were included in the study. Two intervention patients dropped out, leaving a study population of 56 participants (28 in each group) (Figure 1). As far as we know, 14 (50%) of the intervention patients completed the intervention, i.e. discussed the Capacity Note with their manager and returned it to the PHC.

**Table 2.** Stated reasons for declining participation in the study among patients identified as eligible by an intervention or control physician

Reasons for not participating	Patients identified by intervention physician (n)	Patients identified by control physician (n)	Total (n)
Lack of energy	5	5	10
Hesitant to talk to employer	7	1	8
Hesitant to participate in study	2	2	4
Already have a plan for RTW	3	–	3
No reason stated	8	2	10
Total	25	10	35

### Participant characteristics

Six individuals were excluded from the data analysis (see Figure 1) and the final sample consisted of 50 participants: 24 in the intervention group and 26 in the control group. Most participants were women (intervention 71%; control 77%) and worked in unskilled occupations (intervention 57%; control 63%), with no statistically significant differences between groups (Table 3).

**Table 3.** Baseline characteristics of study participants (n = 50) in intervention and control groups, respectively, and analysis of group differences

Baseline characteristics	Intervention (n = 24)	Control (n = 26)	*p*
	n (%)	n (%)	
Gender			0.624
Female	17 (70.8)	20 (76.9)	
Male	7 (29.2)	6 (23.1)	
Age (years)			0.641
19-34	10 (41.7)	8 (30.8)	
35-44	5 (20.8)	8 (30.8)	
45-63	9 (37.5)	10 (38.4)	
Type of occupation†			0.714
Unskilled	12 (57.1)	15 (62.5)	
Skilled	9 (42.9)	9 (37.5)	

†Five individuals (intervention, n = 3; control, n = 2) had missing information on type of occupation.

### Sickness absence

The sickness absence patterns were similar in intervention and control groups, with no statistically significant group differences for any of the outcomes.

#### Time until full return to work

**Figure 3.**
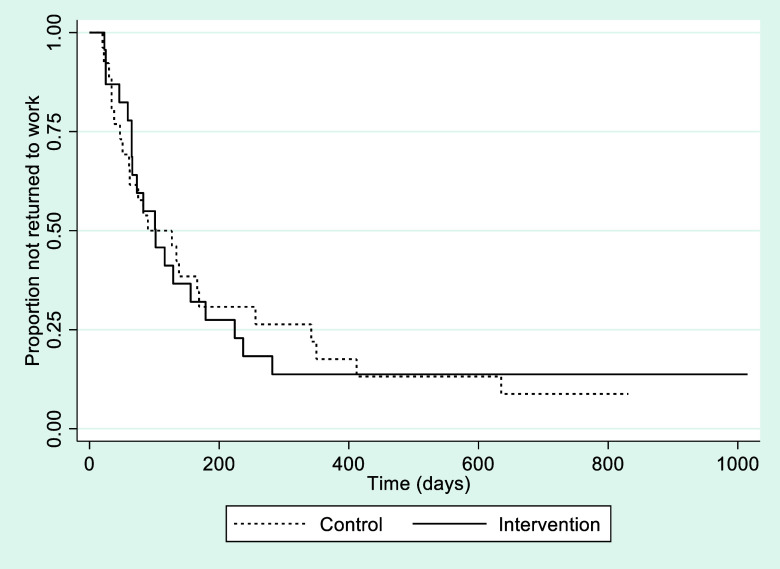
Kaplan–Meier curves for time (days; median) to full return to work in intervention and control groups, respectively.

The median time from inclusion in the study to full RTW was 102 days in intervention group and 90 days in control group (χ^2^ =0.00, df=1, *p*=0.99) (Figure 3).

#### Sick leave status at end of follow-up

In the intervention group, 19 (79.2%) participants were not on registered sick leave at the end of follow-up, while 5 (20.8%) were still on full-time sick leave. In the control group, 22 (84.6%) participants were not on registered sick leave at the end of follow-up, 1 (3.9%) was on part-time sick leave, and 3 (11.5%) were on full-time sick leave.

#### Number of sick leave episodes during follow-up

Most participants had only one sick leave episode during follow-up (intervention, 79%; control, 77%) and the rest had two episodes (intervention, 21%; control, 23%).

#### Number of gross sick leave days at 6, 12 and 17 months after inclusion

No difference was found between the groups in the median number of registered gross sick leave days at 6, 12, and 17 months (Table 4). The median number of days did not increase after 12 months in either of the groups.

**Table 4.** Comparison of registered gross sickness absence days in intervention and control groups at 6 months, 12 months, and 17 months follow-ups

Follow-up	Intervention (n = 24) Median SA days (IQR)	Control (n = 26) Median SA days (IQR)	*p*
6 months	128.5 (72.5–175.5)	122.5 (49.0–178.0)	0.935
12 months	146.5 (78.5–243.5)	127.5 (49.0–327.0)	0.981
17 months	146.5 (78.5–268.0)	127.5 (49.0–357.0)	0.996

SA, sickness absence; IQR, Interquartile range.

## Discussion

This study tested a brief intervention to support communication about work capacity in patients with CMD. We found no differences between intervention and control patients in any of the outcome measures of sickness absence. Despite several measures to facilitate recruitment of patients, our sample was too small. Further studies with larger samples are needed to fully explore the intervention and its effect on sick leave. Some of the lessons learned from this study will be discussed below and can contribute to a more appropriate study design for future studies.

### Recruitment of PHCs

Studies in primary healthcare are usually designed as pragmatic trials. Such trials have higher external validity than randomized controlled trials, which are characterized by high internal validity. However, a drawback of pragmatic trials is that to reach even small significant differences between intervention and controls, the number of participants must be very high (Patsopoulos, 2011). This is admittedly difficult in primary healthcare studies (van der Wouden *et al.*, 2007, Bower *et al.*, 2007). Barriers to recruitment can be related to both the individual GP and the context.

GPs have in previous research expressed their frustration with management of sick leave (Letrilliart and Barrau, 2012) especially in relation to mental health problems (Nilsing *et al.*, 2013), and we received several positive comments about the importance of our study. Still, it was surprisingly difficult to recruit PHCs and engage GPs, reflecting the fact that Swedish primary healthcare is a strained organization with a shortage and high turnover of staff (The Swedish Agency for Health and Care Services Analysis., 2020).

### Recruitment of patients

Recruitment of study participants was slow, and we had to make adjustments and extend the inclusion period. The number of potentially eligible patients at each PHC was lower than expected, being one reason for the slow inclusion rate. In addition, factors such as having a planned contact/visit with the GP during the PHC’s study period played a role. Moreover, as seen in other studies on RTW, recruiting in the early stages of sick leave can be problematic (van Oostrom *et al.*, 2010, Hoefsmit *et al.*, 2016, Keus van de Poll *et al.*, 2020). Patients may have already planned for or started their RTW process, which may reduce motivation to participate/recruit. It is also a period of uncertainty to some degree where both patient and GP might choose ‘watchful waiting’ before more active measures.

We chose short study periods at each PHC to reduce the threshold for PHCs to participate and to keep up motivation among the GPs (Hange *et al.*, 2015). However, it may have led to a lack of confidence and familiarity among GPs regarding study procedures. The rather short information meetings, due to the pressured working situation at participating PHCs, may have further contributed to this. In the end, study periods were longer, but this did not increase the pace of recruitment. We did our best to limit the burden on the GPs. The administrative burden for the users, and the PHCs, was kept to a minimum. Still, the handling of CMD and sick leave is time-consuming, leaving less time for presenting the study, which may have been a barrier for GPs (Arends *et al.*, 2014, Mason *et al.*, 2007). Some GPs also made comments indicating that they found their patients too vulnerable to participate in research, in line with other GPs’ experiences of mental health research in primary care (Mason *et al.*, 2007). Further, it is possible that the subjective assessment of ‘the patient’s readiness to participate’ caused uncertainties among GPs, resulting in asking fewer patients. Arends *et al.*, (2014) found that occupational physicians preferred distinct inclusion criteria and were more willing to recruit patients with a positive attitude and enough energy to participate (Arends *et al.*, 2014). Lastly, there was little pressure on or incentive for the individual GP to engage in the project. Asking the GPs about potential barriers before the study start might have identified further barriers that could have been addressed.

Most of the patients asked to participate accepted, and the drop-out rate was very low, indicating that the intervention was acceptable. Half of the intervention patients completed the intervention, i.e. discussed the Capacity Note with their employer and returned the completed Capacity Note to the GP, which can be considered good given the highly pragmatic approach. Other studies in this patient group and involving several stakeholders have reported much lower adherence (Vlasveld *et al.*, 2013). However, it also suggests that there were barriers to its use. In the qualitative evaluation of the Capacity Note, we found that managers’ lack of time and/or willingness to use the Capacity Note with their employee was one potential barrier (Nordling *et al.*, 2022). Another was the patients’ reluctance to discuss these matters with their manager. The latter was a common reason for not participating in the present study and was expected given the stigma surrounding mental health (Thisted *et al.*, 2020, Dewa *et al.*, 2020, Mangerini *et al.*, 2020). Also, patients were in an early phase of their sick leave, often associated with high levels of stress, and contacting the employer may have been perceived as a too-stressful task at this stage of the process (Noordik *et al.*, 2013).

### Agency as a theoretical departure

The study design emphasized the responsibility of the patient to bridge the lack of information between the GP and the employer. The decision to let the patient take this role was based on agency theory, which claims that people are willing and capable to act and take responsibility in their own lives (Bourdieu, 1977). The intervention aimed to strengthen the patient’s agency and own activity in relation to RTW, which has been described in earlier qualitative studies as important for patients (Mårtensson and Hensing, 2012, Danielsson *et al.*, 2019). Thus, we did not remind or urge patients to contact their employer; it was entirely a decision for the patients. However, it seems that for some patients this was not a suitable approach, they needed more support.

### Sickness absence and RTW in patients with CMD

Systematic reviews on interventions to improve RTW for patients with mental ill-health show that intervention patients, in general, have a faster first RTW but the effect decreases during the first year, meaning that the RTW is not sustainable (Nieuwenhuijsen *et al.*, 2020, van Vilsteren *et al.*, 2015). We chose a long follow-up time to see if RTW was stable. Most participants in the study had returned to work at the end of follow-up and had only one sick leave episode, indicating a sustainable RTW. However, we found no significant differences between the two groups. This is most likely explained by the small sample size. In addition, only half of the intervention patients completed the intervention which makes comparison of the groups more difficult.

### Strengths and limitations

This study took a novel approach by enabling communication between GPs and patients’ employers by engaging the patient. This approach has not, to our knowledge, been tested before. It is also novel in the sense that the intervention is not a set method or guideline, but rather a tool to support the GPs in their own subjective assessment of the patient (Mason *et al.*, 2007, Nordling *et al.*, 2020).

The most important limitation of the study was the small sample size, which prevented us from reaching any conclusions about the effect of the intervention on sickness absence. Another limitation is the risk of contamination. Even though GPs work rather individually they may have discussed the intervention during breaks. This could have been avoided by randomisation at PHC level. Furthermore, it is possible that control physicians gave work-related questions more consideration than usual after being informed about the study and filling in part 1 of the Capacity Note with their patients. These potential sources of bias must be addressed in a future study. Other areas that would need consideration in the design of a new trial are requesting more time for information about the study, optimal length of study periods and level of pragmatism. A more controlled trial might increase recruitment and level of adherence, making an effective evaluation possible, but would to some extent violate the agency approach. Finally, in a controlled trial, the randomization is expected to take care of possible differential distributions of factors of importance for the outcome. In this study, possible factors could be variations in type of CMD, earlier episodes, intensity of symptoms, comorbidity etc. We did not gather detailed information through medical journals or by interviewing participants. In a future study, this could be an advantage even if the sample size is larger.

## Conclusions

This pragmatic trial tested the Capacity Note – a brief intervention to support early communication between GP, patient, and employer to promote RTW among primary healthcare patients with CMD. Due to the small sample size, we could not determine whether the intervention was an effective way to reduce length of sick leave. The recruitment rate was negatively affected by suboptimal research conditions in Swedish primary healthcare. Overall, the intervention seems feasible, but the vulnerability of the patient group calls for individualized assessments. The agency approach may not be suitable for all patients; some patients may need additional support. The findings from this study provide valuable input for any future studies on the current intervention as well as for other studies on stakeholder communication about mental health and work.
